# Effect of Pyrethroids on the Colony Growth and Metabolic Activity of Entomopathogenic Fungi of the *Beauveria* Genus

**DOI:** 10.3390/insects16050533

**Published:** 2025-05-18

**Authors:** Anna Majchrowska-Safaryan, Sylwia Różalska, Cezary Tkaczuk, Monika Nowak

**Affiliations:** 1 Faculty of Agricultural Sciences, Institute of Agriculture and Horticulture, University of Siedlce, 08-110 Siedlce, Poland; anna.majchrowska-safaryan@uws.edu.pl; 2 Department of Industrial Microbiology and Biotechnology, Faculty of Biology and Environmental Protection, University of Lodz, 90-136 Lodz, Poland; sylwia.rozalska@biol.uni.lodz.pl (S.R.); monika.nowak@biol.uni.lodz.pl (M.N.)

**Keywords:** biological methods, entomopathogenic fungi, *Beauveria* genus, pyrethroids, colony growth, metabolic activity

## Abstract

Pyrethroids are chemical insecticides used on a large scale in agriculture, horticulture, and forest protection. In order to reduce their use in IPM, alternative methods of controlling insect pests are introduced, such as the use of biopesticides based on entomopathogenic fungi (EPF). Species of the *Beauveria* genus are characterized by a very broad spectrum of action, which is why they are often used to produce preparations based on EPF. The aim of the study was to determine the effect of different doses of tested pyrethroids on the colony growth and metabolic activity of EPF from the *Beauveria* genus. In vitro, the effect of three pyrethroids (deltamethrin, λ-cyhalothrin, and α-cypermethrin) added to SDA medium at a dose 10 times lower than the recommended field dose (A), the recommended field dose (B), and 10 times higher than the recommended field dose (C) on colony growth and metabolic activity of *B. bassiana* and *B. brongniartii* was tested. The research carried out showed that pyrethroid insecticides used in the experiment showed various toxic effects towards the tested EPF of the genus *Beauveria.* The studies conducted showed that on the 20th day of the observation, λ-cyhalothrin used in the recommended field dose limited the growth of *B. bassiana* to the least extent in relation to the other tested pyrethroids. However, with respect to the fungus *B. brongniartii*, no toxic effect of this pyrethroid was found. Based on the results obtained, it was found that λ-cyhalothrin used in the recommended field dose and 10 times lower than recommended significantly increased the metabolic activity of *B. bassiana*. In relation to the *B. brongniartii* strain, detlamethrin used in each of the tested concentrations significantly affected its viability.

## Simple Summary

The aim of the study was to determine the effect of different doses of tested pyrethroids on the colony growth and metabolic activity of EPF from the *Beauveria* genus. In vitro, the effect of three pyrethroids (deltamethrin, λ-cyhalothrin, and α-cypermethrin) added to SDA medium at a dose 10 times lower than the recommended field dose (A), the recommended field dose (B), and 10 times higher than the recommended field dose (C) on colony growth and metabolic activity of *B. bassiana* and *B. brongniartii* was tested. The studies conducted showed that on the 20th day of the observation, λ-cyhalothrin used in the recommended field dose limited the growth of *B. bassiana* to the least extent in relation to the other tested pyrethroids. However, with respect to the fungus *B. brongniartii*, no toxic effect of this pyrethroid was found. λ-cyhalothrin used in the recommended field dose and 10 times lower than recommended significantly increased the metabolic activity of *B. bassiana*. Deltamethrin used in each of the tested concentrations significantly affected *B. brongniartii* viability.

## 1. Introduction

Biological methods of plant protection against pests in plant production are one of the alternative methods to chemical agents. They are treated as priority actions in IPM (Integrated Pest Management) as well as in the assumptions of the European Green Deal [1,2,3]. However, their implementation requires extensive knowledge of the ecology, biology, and systematics of harmful organisms and their natural enemies, as well as the impact of the synthetic pesticides used [4,5]. One of the most important directions in the development and implementation of biological methods in plant protection is the use of biopreparations based on bacteria, fungi (entomopathogenic fungi EPF) and viruses [1,6,7,8,9].

Entomopathogenic fungi are microorganisms that are used as bioinsecticides in agriculture, especially organic agriculture. Species from the *Beauveria* and *Metarhizium* genera, which are characterized by a very wide spectrum of activity, are often used to produce preparations based on EPF [10,11,12]. The above-mentioned types of EPF not only reduce the number of pests but also endophytically penetrate plant tissues, positively influencing their growth and development, and exhibit properties that allow them to be used in biotechnological processes such as the biosynthesis of nanoparticles [6,10,13,14,15,16]. The main place where EPF occurs is the soil environment and, therefore, they are exposed to contact with pesticides commonly used in plant protection, including pyrethroid insecticides. These microorganisms have developed various adaptive mechanisms that allow them to survive in unfavorable conditions [16,17]. There are 12 EPF strains on the list of active substances approved in the EU. Biopreparations containing EPF are currently used mainly to control the population of thrips, beetles, aphids, whiteflies, and mites in fruit and vegetable crops and ornamental plants [18,19].

Pyrethroids are insecticides commonly used to control arthropods in agriculture, forestry, and greenhouses. They are neurotoxic substances that disrupt the nervous system of insects [16,20,21,22,23]. Due to their widespread use by farmers in plant protection, their residues can be found in all elements of the environment. Despite the fact that pyrethroids are less harmful and safer than other insecticides, it has been proven that they have a negative impact on living organisms [16,24,25].

Studies conducted so far have shown that EPF and pyrethroids can not only be used together to control pests but also coexist in the environment. Therefore, it seems important to not only understand the influence of pyrethroids on the growth of EPF but also on their metabolic activity [16,26,27].

The aim of the study was to determine the effect of different doses of tested pyrethroids on the colony growth and metabolic activity of selected EPF from the *Beauveria* genus.

## 2. Materials and Methods

### 2.1. Fungal Isolates

The effect of preparations containing pyrethroides on EPF colony growth was investigated in laboratory conditions. Two selected species of the *Beauveria* genus were used in the research: the fungus *B. bassiana* (Bals.-Criv.) Vuill. (B03-UPH) isolated from meadow soil collected in Klimonty (Mazowieckie Voivodeship, Poland) and *B. brongniartii* (Sacc.) (B04-UPH) isolated from mid-field woodlots in Boćki (Podlaskie Voivodeship, Poland). The cultures of the fungi used in this experiment were deposited in the fungal collection of the Institute of Agriculture and Horticulture, the University of Siedlce, Poland, and stored in an SDA medium at 4 °C (bioMérieux, Craponne, France). They were identified macroscopically using standard keys [28,29], and their systematic affiliation was also confirmed by molecular identification. The ITS marker, proposed as a universal DNA code marker for fungi, was chosen for identification [30]. Molecular identification of isolates was carried out in the mycological laboratory at the Biological and Chemical Research Center of the University of Warsaw, using Qiagen and Blirt tools (DNA isolation kits, PCR kit and cleaning kit). The PCR reaction was carried out according to the procedure provided by Kovač et al. [31]. Sanger sequencing was used with single ITS2, ITS3, ITS4, and ITS5 primers and the BigDye Terminator Cycle Sequencing Ready Reaction Kit (Applied Biosystems, Waltham, MA, USA), containing fluorescently labeled dideoxynucleotide triphosphates (ddNTPs), deoxynucleotide triphosphates (dNTPs), and Taq-FS polymerase and buffer. After sequencing, product cleaning was conducted by molecular filtration on columns with Sephadex G-50, and the reading of the result was entrusted to Genomed (Warsaw, Poland). The sequences were compared to those available in the NCBI database using the BLASTN 2.2.2 algorithm [32].

### 2.2. The Insecticides Used in the Experiment

Three insecticides from the pyrethroid group were used in the laboratory experiment: Decis Mega 50 EW (Bayer SAS, Puteaux, France), (recommended field dose for wheat protection-0.1–0.125 L/ha, active substance, and its content-deltamethrin 50 g/L), Karate Zeon 050 CS (Syngenta, Warsaw, Poland), (recommended field dose for wheat protection-0.1 L/ha, active substance, and its content λ-cyhalothrin 50 g/L), and Cyperkil max 500 EC (Arysta LifeScience Benelux SPRL, Brussels, Belgium) (recommended field dose for wheat protection−0.05 L/ha, active substance, and its content α-cypermethrin-500 g/L). The pyrethroids selected for the experiment are among the most frequently used insecticides from this group to control insect pests in agricultural and horticultural crops in Poland.

The tested preparations were used in three doses:

A—dose 10 times lower than recommended
B—recommended field dose
C—dose 10 times higher than recommended

The selection the above does of pyrethroids (especially A and C) was guided by the fact that the dose of pesticide that actually reaches the insect or soil in the field conditions, may differ from the recommend one. Similar doses of insecticides were tested in laboratory conditions in the works concerning EPF conducted by other authors [16,20,33,34].

In the experiment, Sabouraud dextrose agar (SDA) produced by bioMérieux was used as a culture medium, with casein enzymatic hydrolyzate-5.0 g, hydrolyzed animal tissues-5.0 g, glucose-40 g, and agar-15.0 g. They were all sterilized using a steam-pressure autoclave at 121 °C under a pressure of 1 atmosphere. The tested insecticides were added to the prepared culture medium in the tested doses. They were then transferred to sterile plastic Petri dishes with a diameter of 90 mm.

### 2.3. Effect on Colony Growth

In the first part of the experiment, the tested fungal isolates were grown on the SDA (bioMérieux, France) medium at 21 ± 1 °C. Before starting the experiment, conidial viability was assessed using the plate count technique on SDA, and viability was >96% and >94% for tested isolates of *B. bassiana* and *B. brongniartii*, respectively. A fragment of mycelium from 10-day-old cultures was sampled with a preparation needle, to be inoculated centrally into the solid SDA medium on Petri dishes (90 mm, Noex, Komorniki, Poland) with the determined dose on pyrethroids. The plates with the inoculated isolates were placed in incubators, protected from light, at 21 ± 1 °C. Colony growth observations were carried out every 5 days until day 20, by measuring the colony diameter in mm. The experiment was performed in four repetitions. The control consisted of cultures grown on substrates without the pyrethroids. The results are presented as the size of the colony diameter expressed in mm and in the case of the 20th day of the culture as a percentage in relation to the control.

### 2.4. Metabolic Activity of Beauveria Strains

Each slant containing the fungal strain was rinsed with 5 mL of SDA (bioMérieux, France) medium and the resulting spore suspension was filtered through a funnel lined with glass wool. The filtrate containing the spores was collected and diluted to a density of 1 × 10⁶ spores/mL. The insecticides were diluted to obtain the following concentrations: A—10 times higher than the recommended dose (concentration A), B—the recommended dose (concentration B), and C—10 times lower than the recommended dose (concentration C). An amount of 100 μL of SDA medium with the test insecticides at concentrations A, B, and C were added to each well of a microtiter plate. Then, 100 μL of the prepared spore suspension was added to each well. At the same time, wells containing biotic samples were prepared according to the scheme above, where 100 μL of fresh SDA medium was added instead of 100 μL of insecticides. All plates were incubated at room temperature for 72 h in the dark. After incubation, 100 μL of freshly prepared fluorescein diacetate solution (2 mg/mL in 0.1 M phosphate buffer, pH 6.4), (Sigma-Aldrich, Taufkirchen, Germany) was added to each well. The plates were then incubated in the dark for 30 min at room temperature. The experiment was performed in four repetitions. Fluorescence was measured at an excitation wavelength of 485 nm and an emission wavelength of 520 nm using an OMEGA spectrofluorimetric reader (BMG Labtech, Offenburg, Germany). The final results were expressed as percentages, with the fluorescence intensity of the biotic control set to 100%. For each tested sample, the fluorescence intensity was calculated relative to this control. Values below 100% indicated a decrease in the measured parameter, while values above 100% indicated an increase.

### 2.5. Statistical Analyses

The results obtained were statistically processed using the Statistica program v. 13.3 (TIBCO Software Inc., Palo Alto, CA, USA). The first stage of the analysis was to check with the chi-square λ^2^ test whether the distribution of the tested trait (colony growth) in the sample followed a normal distribution. Since the data did not have a normal distribution, the transformation y = log (x + 0.5) was applied. Then, on the transformed data, one-way ANOVA analysis was performed for each factor separately according to the following model:

*y_ij_* = *m* + *a_i_* + *e_ij_*

where

- *m* is the population average;
- *y_ij_* is the value of the examined trait (colony growth);
- *a_i_* is the effect of the ith level of factor A (fungal species, dose of preparation, observation time); and
- *e_ij_* is the random error.

When the factor’s effect was significant, the Tukey test was used at α = 0.05 to compare the means (posthoc analysis).

The metabolic activity was analyzed for normality using the Shapiro–Wilk test, and significant differences between treatment groups were determined using a one-way ANOVA followed by Dunnett’s test. The standard deviation was calculated (SD).

## 3. Results

### 3.1. Effect on Colony Growth of EPF

The pyrethroid insecticides used in the experiment showed various toxic effects towards the tested EPF of the genus *Beauveria* (Table 1 and Table 2). Deltamethrin and α-cypermethrin in a dose 10 times higher than recommended field dose (C) on the 5th day of the observation completely inhibited the growth of the *B. bassiana* fungus (Table 1). On days 15 and 20 of observation, α-cypermethrin used in dose C statistically significantly (*p* < 0.012) limited the growth of *B. bassiana* colonies. Among the tested pyrethroids, λ-cyhalothrin used in the recommended field dose (B) at each of the observation dates limited the growth of fungal colonies to the least extent compared to the control, and this difference was statistically significant (*p* < 0.013). On the 20th day of the observation, at a dose 10 times lower than the recommended field dose (A), deltamethrin had the weakest effect on the growth of *B. bassiana* among the tested pyrethroids. This difference was statistically significant (*p* < 0.011) in relation to the control and the other tested doses of the preparation. Karate Zeon 050 CS, containing α-cypermethrin, at dose C showed statistically the strongest (*p* < 0.021) inhibitory effect on the growth of colonies of the tested EPF species. Among the tested pyrethroids, the use of λ-cyhalothrin at the recommended field dose (B) limited the growth of *B. brongniartii* colonies the least (Table 2).

The use of pyrethroids at a dose 10 times lower than the recommended field dose (A) limited the growth of *B. brongniartii* colonies the least, and in the case of λ-cyhalothrin, a slight effect of stimulating colony growth was observed.

**Figure 1:**
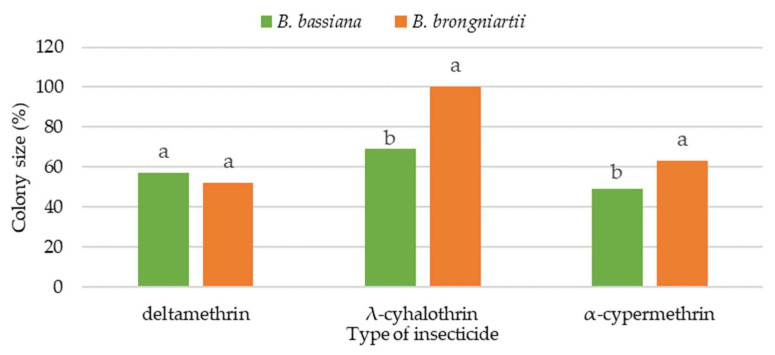
Colony size *B. bassiana* and *B. brongniartii* on the 20th day of growth on media with recommended field dose (B) of tested pyrethroids (% in relation to the control); a,b—means within columns with the same lowercase letters are not significant at α = 0.05.

Comparing the growth of the tested EPFs from the *Beauveria* genus on the 20th day of observation, at the recommended field dose of pyrethroids (B), it was found that deltamethrin had a stronger inhibitory effect on the fungus *B. brongniartii* than on *B. bassiana* (Figure 1). The *B. bassiana* fungus showed statistically significant (*p* < 0.013) greater sensitivity to the presence of λ-cyhalothrin and α-cypermethrin in the culture medium than *B. brongniartii.*

The λ-cyhalothrin used in the recommended field dose did not show any inhibitory effect on the fungus *B. brongniartii*. In relation to both tested EPFs, the pyrethroid λ-cyhalothrin had the least toxic effect, and the fungal colonies reached the largest sizes.

### 3.2. Insecticides Effects on Metabolic Activity of EPF

**Figure 2:**
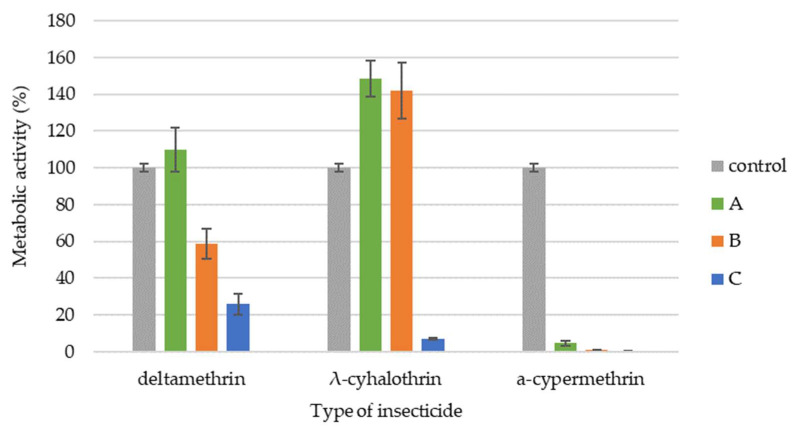
Metabolic activity of *B. bassiana* exposed to different doses of the tested pyrethroids (A—10-fold lower than the recommended field dose; B—recommended field dose; C—10-fold higher than the recommended field dose). Statistically significant differences within each treatment group, compared to the control, were determined using one-way ANOVA followed by Dunnett’s test.

The metabolic activity of *B. bassiana* varied depending on the insecticide and concentration tested (Figure 2). Deltamethrin, at both the recommended field dose and 10 times that concentration, inhibited metabolic activity. The α-cypermethrin exhibited the strongest inhibitory effect, almost completely suppressing metabolic activity. Conversely, λ-cyhalothrin demonstrated a more complex effect. At the recommended field dose and 10 times lower than the recommended dose, λ-cyhalothrin increased *B. bassiana* metabolic activity by approximately 60%. However, a tenfold increase above the recommended dose significantly inhibited metabolic activity. Only a dose 10 times lower than the recommended field dose of deltamethrin resulted in no significant statistical difference (*p* < 0.068) in the metabolic activity of the tested strain compared to the control.

**Figure 3:**
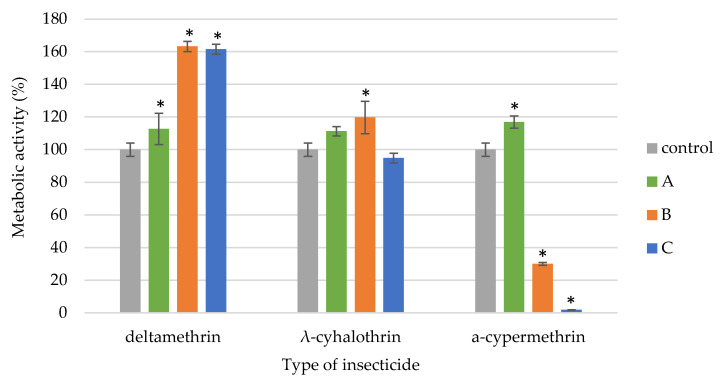
Metabolic activity of *B. brongniartii* exposed to different doses of the tested pyrethroids (A—10-fold lower than the recommended field dose; B—recommended field dose; C—10-fold higher than the recommended field dose). Statistically significant differences within each treatment group, compared to the control, were determined using one-way ANOVA followed by Dunnett’s test and are indicated by * (*p* < 0.05).

The pyrethroids exhibited varying effects on the metabolism of *B. brongniarti* (Figure 3). Deltamethrin significantly increased metabolic activity across all concentrations. The λ-cyhalothrin only enhanced metabolic activity at the recommended field dose while other tested concentrations of the insecticides had no statistically significant (*p* < 0.071) effect on the viability of *B. brongniartii* strain. The α-cypermethrin had a contrasting effect on *B. brongniartii* metabolism. While the recommended field dose and a ten-fold higher concentration inhibited metabolic activity, the concentration 10 times lower than recommended enhanced it.

## 4. Discussion

One of the main factors limiting the use of biopesticides containing entomopathogenic fungi in plant protection is the impact of chemical pesticides on their pathogenicity. Most of laboratory research indicates a negative effect of pesticides on EPF [6,15,35,36,37,38,39]. Pyrethroids are pyrethrin analogs that affect the nervous system of insects by interfering with the activity of chloride and sodium channels [25,40]. They are used in agriculture, forestry, and urban areas and show higher efficacy and lower toxicity compared to other insecticides [24].

Based on the conducted research, it was found that pyrethroids added to the culture medium had a different impact on the growth of the tested EPF of the *Beauveria* genus. The effect of pyrethroid insecticides on the development of colonies of the tested fungi depended on the type of active substance used, the concentration of pyrethroid in the culture medium, and the fungus species. In vitro studies conducted so far have shown that individual fungal species, and sometimes even individual strains within a species, are characterized by varying sensitivity to plant protection products [6,16,27,41,42]. Studies show that entomopathogens introduced into the environment (interchangeably with synthetic insecticides) as part of IPM can maintain their activity in the soil environment for many years, effectively reducing the pest population [43].

When interpreting the results obtained, it should be taken into account that tests were conducted in laboratory conditions which do not always correspond to the situation in the field both in direct contact with the sprayed insects and in the soil environment. The overall actual effect of the pesticides tested on the populations and performance of insect pathogenic fungi in the field is difficult to evaluate. It is certain that most of the time the concentrations of the chemicals that reach the fungi in the soil are smaller than those employed in our experiment. Locally, however, the concentrations may be much higher and exceed the normal ones. This may be especially true for pesticides that are applied directly into the soil. On the other hand, fungi growing on agar in Petri dishes are grown under optimum conditions, which may make them more tolerant to the chemicals than in nature where suboptimal growing conditions, many different antagonists and adverse weather conditions prevail. In such situations even a small additional stress factor may significantly lower their fitness and performance. On the other hand, pesticides are affected and degraded by different environmental factors on the plant and in the soil [41,44].

In the literature, the results of studies conducted so far indicate a negative impact of insecticides on EPF used to control crop pests. Amutha et al. [38] in their study showed that profenofos and methyldemeton inhibited the growth of *B. bassiana* by 93.72% and 95.17% after 14 days of inoculation and by 83.55% and 93.86% after 30 days. The addition of indoxacarb to the culture medium inhibited the growth of *B. bassiana* colonies by 100% after 14 and 30 days of inoculation. Forlani et al. [26] in their study showed that deltamethrin used at a concentration of 40 lg cm^−2^ caused a significant decrease in the number of colonies forming units of *B. bassiana*. Litwin et al. [16] found that pyrethroids used at recommended (and higher) doses strongly inhibited the development of *B. bassiana* during the initial growth period, reducing the rate of colony formation by more than 40%, whereas at lower doses, these insecticides had minimal inhibitory effect. Bednarek et al. [45] in their study found that the insecticides Karbosulfan inhibited, while Karbofuran stimulated the growth of *B. brongniartii* fungus colonies. Widyaningsih et al. [39] studied the effect of Profenofos pesticide and λ-cyhalothrin on the growth of EPF isolated from citrus orchard and found that all EPF (*Hirsutella* sp., *Paecilomyces* sp., *B. bassiana* and *M. anisopliae*) used in this experiment did not show significant growth reduction after treatment with different doses of λ-cyhalothrin pesticide. The use of the tested pyrethroids in higher concentrations can significantly limit the growth of EPF isolates [6,39]. Srinivasulu and Ortiz [46] in their studies showed that with the use of higher concentrations of pesticides, the fungal population gradually decreased and reached a minimum concentration of 10 kg ha^−1^. This suggests that the concentration of pesticides used in the field or in the substrate must be controlled. In contrast, studies conducted by James and Elzen [47], Bhattacharya et al. [48], and Kumar et al. [49] showed that *B. bassiana* strains were highly compatible with the insecticides imidacloprid and spinosad, showing no inhibition of growth, sporogenisation, or viability.

Our own studies showed that the metabolic activity of *B. bassiana* and *B. brongniartii* varied depending on the insecticide and the tested concentration. Studies conducted so far indicate that insecticides commonly used in agricultural production not only limit growth but also cause abnormalities in cellular metabolic pathways and damage EPF cell structures [16,25,50,51,52,53]. Litwin et al. [16] showed that the tested pyrethroids did not show a negative effect on the metabolic activity of *B. bassiana*, and even a slight increase in activity was found in samples exposed to the tested substances. In 24 h cultures, a 1.47-fold increase in metabolic activity was noted in the presence of deltamethrin, a 1.36-fold increase in the presence of λ-cyhalothrin, and a 1.32-fold increase in the presence of α-cypermethrin. In 36 h cultures, a 1.1-fold increase in metabolic activity was observed in the presence of λ-cyhalothrin, while in the presence of α-cypermethrin and deltamethrin, the metabolic activity in the samples was comparable to that observed in the control. In 48 h cultures, no differences in metabolic activity were found between cultures exposed to pyrethroids and biotic control. Różalska et al. [50] examined the metabolic activity of *Metarhizium robertsii* and found that the addition of both 4-n-nonylphenol and technical nonylphenol caused a statistically significant decrease in the metabolic activity of the tested strain. Litwin et al. [25] found that the soil EPF *B. bassiana* ARSEF 2860 accumulated pyrethroids as early as day 2 of incubation with an average efficiency of 90%. Pyrethroids accumulating in large amounts in the mycelium of *B. bassiana* induced oxidative stress and interacted differently with enzymes of basic metabolic pathways. This study showed that accumulated pyrethroids reduced phospholipase C activity, which increased the triacylglycerol to diacylglycerol (TAG/DAG) ratio, especially in the mycelium in which α-cypermethrin had accumulated.

## 5. Conclusions

Entomopathogenic fungi have a broad spectrum of action and can infect a wide range of arthropod species including crop pests. Research shows that in addition to the use of EPF in biocontrol, these fungi can also be used for removing harmful substances from the environment, because they possess enzymes that enable the removal of toxic compounds of anthropogenic origin. The results of our research confirm that the use of pyrethroids affects the colony growth and metabolic activity of the fungi *B. bassiana* and *B. brongniartii*. The studies conducted showed that on the 20th day of the observation, λ-cyhalothrin used in the recommended field dose limited the growth of *B. bassiana* to the least extent in relation to the other tested pyrethroids. However, with respect to the fungus *B. brongniartii*, no toxic effect of this pyrethroid was found, and even a slight stimulation of its growth was observed at a dose 10 times lower than the recommended one. Based on the results obtained, it was found that λ-cyhalothrin, used in the recommended field dose and 10 times lower than recommended, significantly increased the metabolic activity of *B. bassiana*. In relation to the *B. brongniartii* strain, deltamethrin used in each of the tested concentrations significantly affected its viability. Regardless of the pyrethroid used, at a dose 10 times higher than the recommended field dose, it was found that the *B. brongniartii* fungus showed greater viability compared to the *B. bassiana* strain. This indicates that the tested *B. brongniartii* strain can potentially be used interchangeably with synthetic pyrethroids. Data obtained in this study may guide future recommendations of use pyrethroids in IPM programs where *B. bassiana* and *B. brongniartii* are intended to be used as a biocontrol agent of insect and mite pests in agricultural and horticultural crops.

## Figures and Tables

**Table 1:** Diameters of *B. bassiana* fungus colonies during cultivation on media with the addition of selected insecticides (expressed in mm).

Insecticide/Active Substance	Dose	Diameter of Fungus Colony (mm)			
		5th Day	10th Day	15th Day	20th Day
DECIS Mega 50 EW (deltamethrin)	A	7.3 ± 0.47 b	18.3 ± 1.24 a	25.6 ± 1.24 a	33.3 ± 1.84 b
	B	3.3 ± 0.47 c	9.4 ± 1.24 b	17.0 ± 1.50 b	25.0 ± 0.81 c
	C	n.g. ± 0.0 d	4.7 ± 1.69 c	14.6 ± 2.49 b	19.6 ± 1.24 d
Control		10.5 ± 0.57 a	20.3 ± 0.50 a	28.0 ± 0.81 a	44.0 ± 1.41 a
KARATE ZEON 050 CS (λ-cyhalothrin)	A	9.6 ± 0.47 a	19.3 ± 0.48 a	24.6 ± 1.25 b	31.3 ± 1.25 b
	B	7.6 ± 0.48 b	17.7 ± 0.47 b	23.6 ± 0.49 b	30.3 ± 0.48 b
	C	3.7 ± 0.94 c	8.6 ± 0.94 c	17.3 ± 1.33 c	23.6 ± 1.42 c
Control		10.5 ± 0.57 a	20.3 ± 0.50 a	28.0 ± 0.81 a	44.0 ± 1.41 a
CYPERKIL MAX 500 EC (α-cypermethrin)	A	9.2 ± 0.94 ab	18.6 ± 0.47 a	23.3 ± 0.47 b	32.0 ± 0.81 b
	B	7.3 ± 1.70 b	11.5 ± 1.25 b	16.4 ± 0.47 c	21.6 ± 0.94 c
	C	n.g. ± 0.0 c	5.3 ± 1.70 c	8.6 ± 0.94 d	12.6 ± 0.94 d
Control		10.5 ± 0.57 a	20.3 ± 0.50 a	28.0 ± 0.81 a	44.0 ± 1.41 a

A—dose 10 times lower than recommended; B—recommended field dose; C—dose 10 times higher than recommended; abcd—means within columns with the same lowercase letters are not significant at α = 0.05; ±standard deviation; n.g.—no growth.

**Table 2:** Diameters of *B. brongniartii* fungus colonies during cultivation on media with the addition of selected pyrethroids (expressed in mm).

Insecticide/Active Substance	Dose	Diameter of Fungus Colony (mm)			
		5th Day	10th Day	15th Day	20th Day
DECIS Mega 50 EW (deltamethrin)	A	5.0 ± 0.0 b	17.6 ± 1.52 b	25.6 ± 0.45 b	31.6 ± 2.88 b
	B	2.3 ± 0.47 c	7.6 ± 0.47 c	15.0 ± 1.73 c	23.3 ± 2.88 c
	C	n.g. ± 0.0 d	4.3 ± 0.52 d	9.6 ± 0.23 d	15.3 ± 1.50 d
Control		13.0 ± 0.81 a	28.3 ± 0.95 a	36.7 ± 0.95 a	45.0 ± 3.74 a
KARATE ZEON 050 CS (λ-cyhalothrin)	A	12.6 ± 0.57 a	26.2 ± 1.52 ab	31.7 ± 2.88 b	46.0 ± 4.35 a
	B	11.6 ± 0.57 a	24.6 ± 1.52 b	33.3 ± 2.88 ab	45.0 ± 3.00 a
	C	2.6 ± 1.15 b	8.6 ± 1.25 c	13.5 ± 1.15 c	19.7 ± 1.52 b
Control		13.0 ± 0.81 a	28.3 ± 0.95 a	36.7 ± 0.95 a	45.0 ± 3.74 a
CYPERKIL MAX 500 EC (α-cypermethrin)	A	11.0 ± 1.00 a	20.6 ± 2.08 b	28.5 ± 2.08 b	37.0 ± 1.73 b
	B	6.0 ± 0.57 b	15.5 ± 1.15 c	21.3 ± 1.52 c	28.3 ± 2.88 c
	C	2.0 ± 0.0 c	4.0 ± 0.0 d	6.6 ± 0.57 d	10.2 ± 0.52 d
Control		13.0 ± 0.81 a	28.3 ± 0.95 a	36.7 ± 0.95 a	45.0 ± 3.74 a

A—dose 10 times lower than recommended; B—recommended field dose; C—dose 10 times higher than recommended; abcd—means within columns with the same lowercase letters are not significant at α = 0.05; ±standard deviation; n.g.—no growth.

## Data Availability

The original contributions presented in this study are included in the article. Further inquiries can be directed to the corresponding author.
